# Step-up approach for the treatment of infected necrotising pancreatitis: real life data from a single-centre experience with long-term follow-up

**DOI:** 10.1186/s12876-024-03289-6

**Published:** 2024-06-28

**Authors:** Claire Valentin, Guillaume Le Cosquer, Géraud Tuyeras, Adrian Culetto, Karl Barange, Pierre-Emmanuel Hervieu, Nicolas Carrère, Fabrice Muscari, Fatima Mokrane, Philippe Otal, Barbara Bournet, Bertrand Suc, Louis Buscail

**Affiliations:** 1 Department of Gastroenterology and Pancreatology, Toulouse Rangueil University Hospital, 1 avenue Jean Poulhès, TSA 50032, Toulouse Cedex 9, 31059 France; 2 Department of Digestive Surgery, Toulouse Rangueil University Hospital, Toulouse, France; 3 Department of Hepatology, Toulouse Rangueil University Hospital, Toulouse, France; 4 Department of Radiology, Toulouse Rangueil University Hospital, Toulouse, France

**Keywords:** Acute pancreatitis, Infected necrosis, Walled-off necrosis, Step-up approach, Lumen-apposing metal stent, Necrosectomy

## Abstract

**Background:**

About 20% of patients with acute pancreatitis develop a necrotising form with a worse prognosis due to frequent appearance of organ failure(s) and/or infection of necrosis. Aims of the present study was to evaluate the “step up” approach treatment of infected necrosis in terms of: feasibility, success in resolving infection, morbidity of procedures, risk factors associated with death and long-term sequels.

**Methods:**

In this observational retrospective monocentric study in the real life, necrotizing acute pancreatitis at the stage of infected walled-off necrosis were treated as follow: first step with drainage (radiologic and/or endoscopic-ultrasound-guided with lumen apposing metal stent); in case of failure, minimally invasive necrosectomy sessions(s) by endoscopy through the stent and/or via retroperitoneal surgery (step 2); If necessary open surgery as a third step. Efficacy was assessed upon to a composite clinical-biological criterion: resolution of organ failure(s), decrease of at least two of clinico-biological criteria among fever, CRP serum level, and leucocytes count).

**Results:**

Forty-one consecutive patients were treated. The step-up strategy: (i) was feasible in 100% of cases; (ii) allowed the infection to be resolved in 33 patients (80.5%); (iii) Morbidity was mild and rapidly resolutive; (iv) the mortality rate at 6 months was of 19.5% (significant factors: SIRS and one or more organ failure(s) at admission, fungal infection, size of the largest collection ≥ 16 cm). During the follow-up (median 72 months): 27% of patients developed an exocrine pancreatic insufficiency, 45% developed or worsened a previous diabetes, 24% had pancreatic fistula and one parietal hernia.

**Conclusions:**

Beside a very good feasibility, the step-up approach for treatment of infected necrotizing pancreatitis in the real life displays a clinico-biological efficacy in 80% of cases with acceptable morbidity, mortality and long-term sequels regarding the severity of the disease.

## Background

Acute pancreatitis (AP) is a common disease, with an increasing incidence [1]. Mortality depends on the severity of AP with two main prognostic factors: organ failure (OF) and the presence of necrosis [2, 3]. Necrotising AP mortality is estimated at around 15% and increases up to 35% in case of infection [3–5]. An interventional procedure is most often necessary in case of infected necrosis. This procedure must be carried out at best at the stage of so called walled-off necrosis (WON). Previously, the standard treatment for severe post-AP necrosis infection was open laparotomy surgery for necrosectomy. Nevertheless, it was associated with high mortality (11–39%) and morbidity (34–95%) [3–5]. We and others developed minimally invasive radiological, endoscopic and surgical techniques [6] that can be included in the step-up approach and appeared finally superior to open surgery with significantly lower new-onset of OF and mortality as well as shorter hospital stay [7–12]. Moreover, the endoscopic approach (when possible) may be superior to minimally invasive surgical ones in term of mortality, major complications and sequels [13–16]. Considering these results, the “step up” approach was validated by an international consensus in 2012 and by other nationals and European consensus [17–20]. The full protocol includes a first step that consists in draining the necrotic cavity either by endoscopic or radiological routes. If drainage is not sufficient to control sepsis and/or OF, the second step is to perform one or more minimally invasive necrosectomy sessions by endoscopy and/or surgery. Finally, in case of failure, the third step is invasive surgery by laparotomy for necrosectomy. Most of the studies analysed the different techniques separately or compared the techniques with each other. In addition, long-term complications and sequels are poorly documented. The objectives of our study were to evaluate this “step up” approach in the real life in terms of: feasibility, success in resolving infection, morbidity of procedures, risk factors associated with death, long-term complications and sequels.

## Methods

### Patients

This observational retrospective monocentric study was conducted in the digestive department of Toulouse University Hospital. The presence of infected necrosis was suspected upon clinical (fever, systemic inflammatory response syndrome (SIRS), appearance or persistence of one or more OF) and/or biological (inflammatory syndrome) and/or radiological arguments (presence of air bubbles within necrotic areas). Necrosis infection was qualified as proven when bacteriological and/or mycological analyses revealed germs in necrosis samples. The OF were defined as previously described [8, 21]: Respiratory failure: PaO2 < 60 mmHg or need mechanical ventilation; circulatory failure: systolic blood pressure < 90 mm Hg despite adequate fluid resuscitation or need for inotropic catecholamine support; renal failure: creatinine level greater than 177 micromol/L or need for hemofiltration or hemodialysis.

Were excluded from the study patients: with pseudocyst, with necrotising AP but without criteria of “suspected or proven necrosis infection” and that already underwent interventional procedure in another centre.

### Study design

**Fig. 1:**
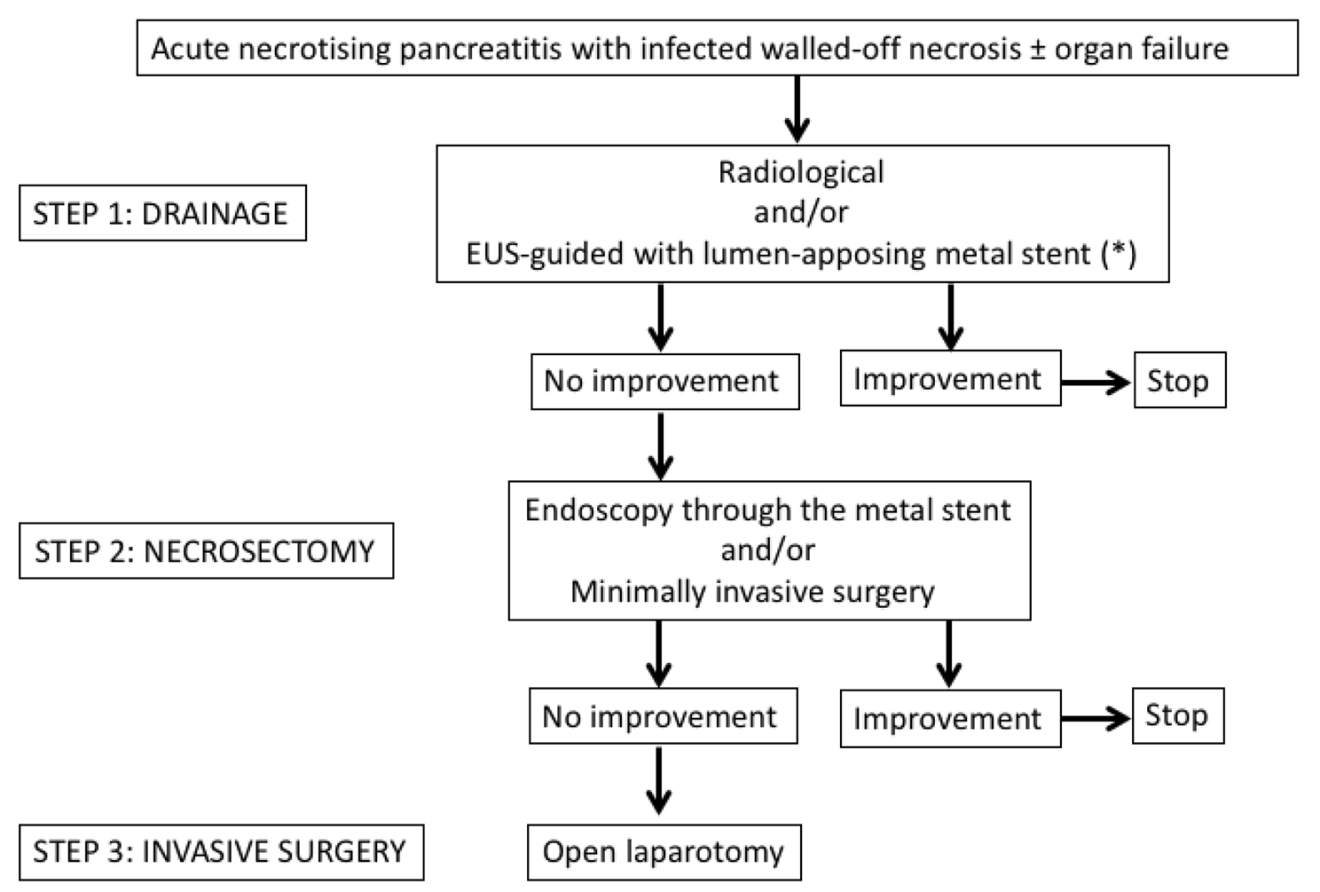
Algorithm of the Step-up approach that has been applied in the present study for the treatment of infected necrotising pancreatitis at the stage of walled-off necrosis

Since the 2012 consensus, our centre had systematically managed necrosis infection using the “step-up” approach [17] as shown in Fig. 1. Treatment decisions were made in a collegial manner after multidisciplinary meeting between gastroenterologists, surgeons, radiologists and practicians of the intensive care department. Failure was defined in this study as: lack of sepsis control (persistent fever, stable or increased biological inflammatory syndrome) and/or persistent OF.

### Objectives of the study and evaluation criteria

The objectives were: (i) feasibility of the step-up approach upon the successful implementation of the strategy at the technical and organisational levels; (ii) efficacy on infection resolution assessed upon to a composite criterion i.e.: resolution of OF (if present initially) and at least two of the following clinical and biological criteria: apyrexia, decrease of at least 50% of CRP serum level, decrease of at least 25% of total leucocytes count; (iii) immediate and delayed (within one month) morbidity of the endoscopic, radiological, and surgical procedures; (iv) 6-months mortality including its risk factors; (v) long term (> 6 months) complications and sequels.

### Procedures of drainage

#### **Radiology**

Radiological drainage was performed (mostly by retroperitoneal routes) using the technique of Seldinger or by direct introduction of a trocar. Fluid was systematically aspirated and immediately inoculated on both aero-anaerobic and mycological culture media. The drains left in place were of the double-port type with a minimum gauge of 10 F and a maximum gauge of 15 F. The necrotic cavity was irrigated with saline 3 to 4 times a day, with recovery by gravity. Depending on the abundance, number and communicating nature of the collections, several drains could be implemented.

#### **Endoscopy**

A transgastric expanding covered lumen apposing meta stent (LAMS − 2 to 3 cm long and 16 mm in diameter – Niti-S™ NAGI™ Stent, Taewoong MedicalⓇ, Korea and since 2016 Axios^™^ system -Boston ScientificⓇ, USA − 2 cm long, 15 mm large) was placed in a single procedure under full endoscopic ultrasound (EUS) control. The procedure began with a puncture using a 19 G needle (Cook MedicalⓇ, Ireland) to aspirate fluid from the necrotic cavity (immediately inoculated for analysis on aerobic-anaerobic and mycological culture media). A guide wire (Jagwire 0.35 mm in diameter, Boston ScientificⓇ, USA) was then wound into the necrotic cavity and let in place after withdrawing the needle. A 10 F cystotome (Cook MedicalⓇ, Ireland) was mounted onto the guide-wire to enlarge the orifice and fit the LAMS under endoscopic and fluoroscopic control. Once the LAMS was in place, a large bore drain (10 F) was usually left in place in the necrotic cavity (through the stent) for daily lavage of the fluid and necrosis for 2 or 3 days. The LAMS was left in place for 4 to 6 weeks before its endoscopic extraction. In some cases, the drainage was done by putting in place within the necrotic cavity two double pig-tail stents (7 to 10 F) by the same initial procedure. This procedure could also precede the subsequent placement of LAMS.

The choice of either radiologic or endoscopic route for drainage was not randomized and was guided by both the localization and the size of the WON collection.

### Procedures of necrosectomy

#### **Endoscopy**

Necrosectomy was performed using a gastroscope penetrating within the necrotic cavity via the LAMS. The necrosis was first washed by an irrigation system, then the debridement and extraction of necrosis were performed step by step using basket snare, foreign body forceps or a diathermic loop as described [6]. One to three sessions were offered depending on the resolution or not of infection. Necrosectomy was considered effective when the necrotic cavity was reduced size and no longer contained infected necrosis but “clean” granulation tissue.

#### **Surgery**

The two minimally invasive surgical techniques were minimal access retroperitoneal pancreatic necrosectomy and videoscopic-assisted retroperitoneal debridement. The approach could be directed by placing a radiological drain the day before the operation to “show the way” to the infected necrotic cavity. Necrotic debris were evacuated by the surgeon’s hand, but also by washing and aspiration. The procedure ended with the placement of a very large-calibre drain to wash out the necrotic cavity (Davol®-drain), allowing both abundant washing of the cavity and aspiration/drainage.

In case of failure, open surgery with median laparotomy and necrosectomy was performed (3rd step), combining lavage of the peritoneal cavity, multiple drainages and often insertion of a laparostomy with parietal suction drainage.

All these procedures have been associated with nutritional assistance, treatments of OF if present, antibiotics and antifungal treatments adapted to antibiograms, and multiple suitable cares for patients hospitalised in intensive care. A subsequent cholecystectomy was performed in case of biliary AP.

### Data collected

We collected all clinical, biological and radiological data before and during the step-up approach: age, gender, main comorbidities, past history of AP, etiology, presence of SIRS and OF, intensive care required, Balthazar score at CT-scan, mean size of the largest collection, patient referred from another center, previous antibiotic treatment, serial mean CRP level, serial mean leucocytes level, mean albumin level, proven infection of necrosis, parenteral nutrition. We also analysed all the characteristics and complications of the interventional procedures. The long-term follow-up was set up to collect possible sequels and comprised at least one visit every 6 months during the first two years and one visit per year thereafter including clinical examination, biology and if necessary CT-scan.

### Statistical analyses

The results are expressed as means ± standard deviation (SD). Qualitative data were compared using the *exact Fisher test* and the *Chi2 test* (with Yates correction), quantitative data were compared with *Student t-test* and *paired Mann-Withney test.* The 6-months death factors were analysed in uni- and multivariate using the logistic regression model. Throughout the study, a *p* > 0.05 was considered statistically significant. The software used were: InStat V°4.0, GraphPad Prims V° 6.0a and Stata.

### Ethical considerations

The study was conducted in accordance with the principles of good clinical practice and the declaration of Helsinki at all times. According to the French ethic and regulatory law (modified French Data Protection Act of January 6, 1978, and the General Data Protection Regulation) retrospective studies based on the exploitation of usual care data do not require submission to an ethical committee and do not require informed written or verbal consent to participate to the research. The retrospective studies have to be declared or covered by reference methodology of the French National Commission for Informatics and Liberties (CNIL). A collection and computer processing of personal and medical data was implemented to analyze the results of the research. After evaluation and validation by the data protection officer and according to the General Data Protection Regulation, the present study completing all the criteria, and it is covered by the MR-004 (CNIL number: 2,206,723 v 0).

## Results

### Patients characteristics

The study included 41 consecutive patients from September 2013 to December 2017. The characteristics of the population are detailed in Table 1.

**Table 1:** Patients characteristics and demography (*n* = 41)

**Demography and medical history** ^**a**^	
Mean age (SD) [range]	60.2 (15) [15–85]
Gender, *n* (%)	
- Men	31 (75.5%)
- Women	10 (24.5%)
Comorbidities, *n* (%)	
- Cardio-vascular	8 (19.5%)
- Pulmonary	7 (17%)
- Kidney	0 (0%)
- Obesity (i.e. BMI ≥ 30)	10 (24.5%)
- Diabetes	2 (5%)
- Tobacco	5 (12%)
Past history of AP, *n* (%)	11 (27%)
Characteristics at the first week after admission^a^	
Etiology, *n* (%)	
- Biliary	25 (61%)
- Alcohol	5 (12%)
- Others^b^	11 (27%)
SIRS, *n* (%)	19 (46.5%)
Organ failure, *n* (%)	14 (34%)
- one	9 (22%)
- multiple	5 (12%)
Intensive care unit, *n* (%)	18 (44%)
Balthazar score, *n* (%)	
- D	4 (10%)
- E	37 (90%)
Referred from another centre, *n* (%)	31 (75.5%)
Previous antibiotic treatment, *n* (%)^c^	28 (68.2%)
Characteristics before step 1 (one day before and/or the day of the procedure)	
Mean time onset of AP and step 1 (days) (SD) [range]	40.2 (26) [18–160]
Organ failure at step 1, *n* (%)	8 (19.5%)
- one	5 (12%)
- multiple	3 (7.5%)
Intensive care unit, *n* (%)	9 (22%)
Mean CRP (mg/l) (SD) [range]	209.4 (103) [18–426]
Mean leucocytes count (/mm3) (SD) [range]	14,354 (8,579) [3,600–35,600]
Mean albumin level (g/l) (SD) [range]	22.4 (6.3) [12–44]
Mean size of the largest collection (cm)	15.1 (3.5) [8–25]
Fine Needle Aspiration before step 1, *n* (%)	6 (14.5%)
Proven Infection of Necrosis, *n* (%)	38 (92.5%)
- bacteria only	23 (60.5%)
- fungal only	3 (8%)
- both	12 (31.5%)
Parenteral Nutrition, *n* (%)	35 (85.5%)

a. Data from patients both directly referred to our center and those secondary referred from another center

b. Idiopathic: 8 (19.5%); Post Endoscopic Retrograde Cholangio Pancreatography (ERCP): 2 (5%); Ischemic: 1 (2.5%)

c. Probabilistic *n* = 18; documented *n* = 10 (staphylococcus aureus *n* = 6 ; E-coli *n* = 3 ; pneumococcus *n* = 1)

### Procedures, patients distribution and outcomes among each step

**Fig. 2:**
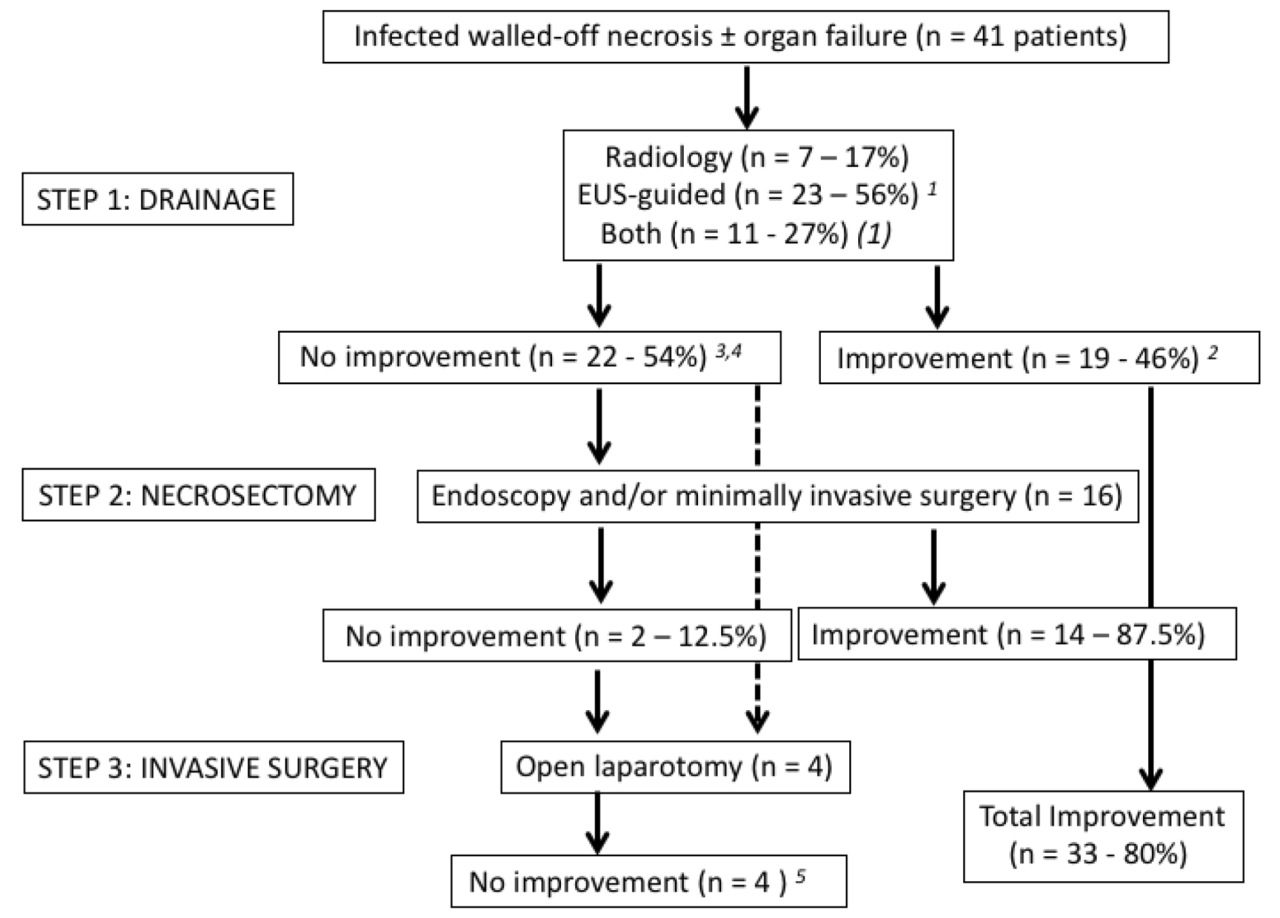
Algorithm of the Step-up approach for the treatment of infected necrotising pancreatitis with: type of drainage and necrosectomy procedures, number of patients per procedure and improvement or not of the infection. 1: Thirty-two patients with lumen-apposing metal stent and 2 patients with double pig-tail stents; 2: Improvement was assessed by a composite criterion: resolution of organ failure(s) and at least two of the following clinical and biological criteria: apyrexia, decrease of at least 50% of CRP serum level, decrease of at least 25% of total leucocytes count; 3: Four patients died at this step; 4: Two patients went directly to the third step due to gravity of the disease; 5: The four patients died

Number and details of interventional procedures at each phase of the step-up approach are given in Table 2. Distribution of patients along the 3 steps is illustrated in Fig. 2 including number of patients with improvement of infected necrosis. Seventeen patients out of 41 (39%) required the passage to step 2: 11 (69%) had one or more endoscopic necrosectomy sessions, 4 (25%) had one or more minimally invasive surgical necrosectomy sessions and 1 (6%) had both. We compared the characteristics of patients that did not required necrosectomy (i.e. patients in which first step was effective versus those that underwent necrosectomy at step 2): among the following factors such as “SIRS, obesity, biliary AP, OF, size of the largest collection and CRP level” only OF before step-up approach (Fisher’s exact test – 17% versus 5% - *p* = 0.028) and the size of the collection (16.4 ± 3.8 cm versus 14 ± 2.9 – unpaired t test – *p* = 0.027) were statistically significant in the “step 2 group”.

**Table 2:** Details of interventional procedures at each phase of the step-up approach for treatment of infected necrotising pancreatitis

STEP 1: Drainage *n* = 41 (100%)	
Endoscopy *n* = 34	
Mean time between onset of AP and stenting: 45.5 days (SD: 29.9) [Range: 18–160]	
EUS guidance: *n* = 34 (100%)	
Type of prosthesis * Lumen-apposing metal stents: *n* = 32 * Double pig tail plastic stents *n* = 6 (alone *n* = 2, before LAMS: *n* = 4)	
Success of stent placement: *n* = 33 (97%)	
Naso-cystic (10 F) drain let in place followed by wash-drainage *n* = 25 (73.5%)	
Mean time of the procedure (min): 37 (SD: 8)	
Radiology *n* = 18 (44%)	
Mean time between onset of AP and drain placement: 47.5 days (SD: 28) [Range: 20–104] (diameter of drains from 10 to 16 French)	
Routes: - Retro-peritoneal: *n* = 15 - Trans-peritoneal: *n* = 6 (3 patients had both retro- and transperitoneal drains)	
Mean number of drains per patient: 2.1 (SD: 1) (Range: 1 to 4)	
STEP 2: Mini-invasive necrosectomy *n* = 16 (39%)	
Endoscopy *n* = 12	
Mean number of procedures per patient: 1.4 (Range: 1 to 3)	
Mean time between stenting and necrosectomy: 16 days (SD: 11)	
Surgery *n* = 5	
Routes: - Retro-peritoneal *n* = 5 - Trans-peritoneal *n* = 0	
Mean number of procedures per patient: 1.2 (Range: 1 to 2)	
Mean time between stenting and surgical necrosectomy: 52 days (SD: 12)	
STEP 3: Open laparotomy for necrosectomy *n* = 4 (9.5%)	

Finally, only 4 patients required the transition to step 3 with a surgical necrosectomy by laparotomy. Unfortunately, these four patients died at least one week after surgery. These four patients had a severe clinical presentation with OF and sepsis as well as multiorgan assistance. Two of them were proposed for surgery immediately after drainage and the two others were not improve by minimal invasive necrosectomy (Fig. 2).

On the whole 8 patients died (19.5% − 4 patients during the first step and 4 during after step 3).

### Feasibility

The “step-up” strategy was implemented for all patients in our centre, making the strategy 100% feasible.

### Effectiveness in resolving infection

The “step up” strategy allowed the infection to be resolved, according to a composite criterion, in 33 patients (80.5%): 19 patients after step 1 and 14 patients after step 2 (Fig. 2). The mean time to resolve the infection was 33.7 days (SD: 25).

**Fig. 3:**
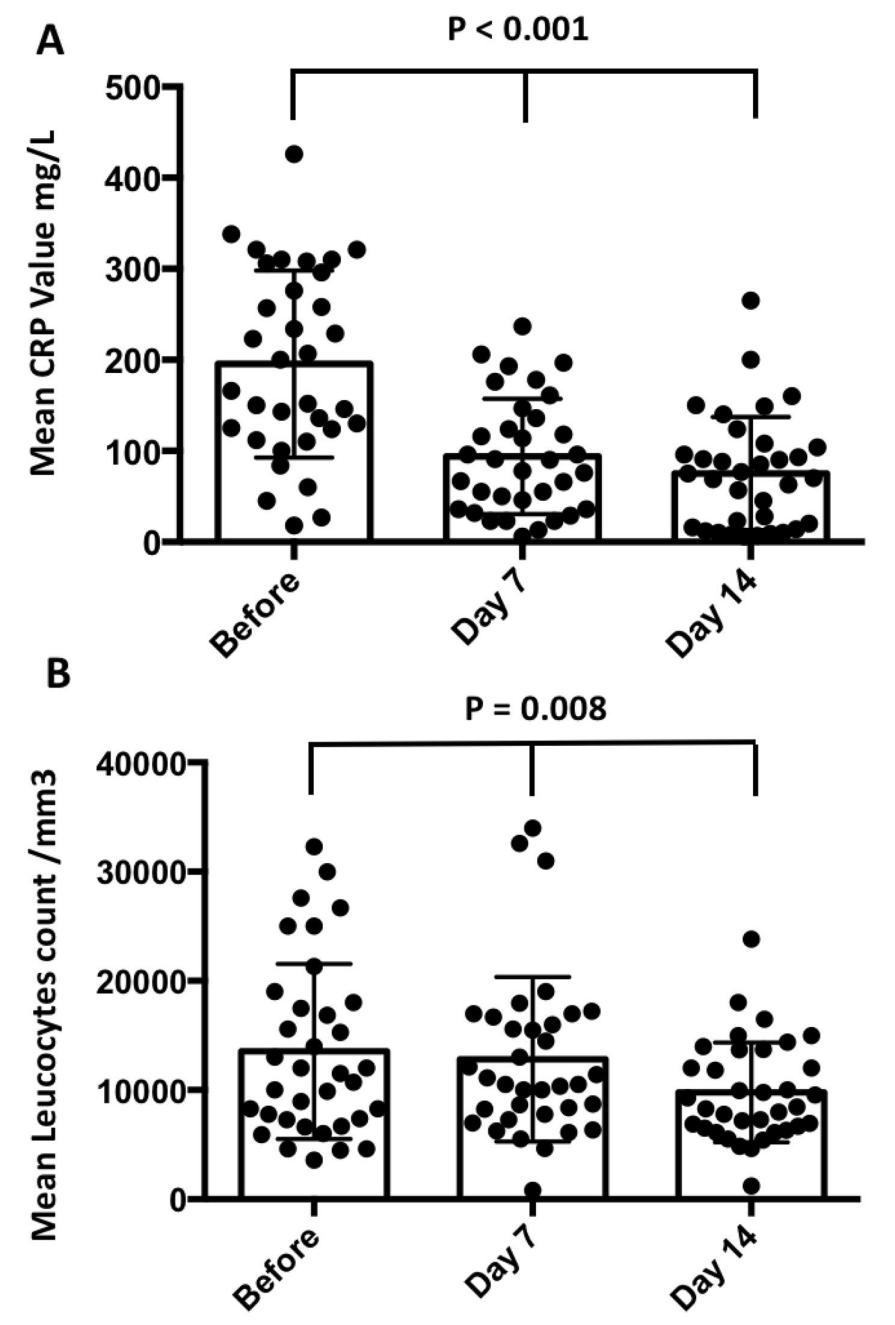
CRP levels (panel **A**) and white blood cell count (panel **B**) before and 7 and 14 days after the first procedure of drainage of walled-off necrosis. 35 patients were investigated for whom we had all dosages before drainage and thereafter at 7 and 14 days after drainage (paired Mann-Whitney test)

We also compared the mean CRP (mg/l) level (Fig. 3A) and leukocyte (/mm3) counts (Fig. 3B) before, 7 and 14 days after the first drainage procedure. We observed a significant decrease of both parameters. On the whole, this first step achieves near to 50% of infection resolution (46%). The synthesis of the results including all evaluation criteria are summarised in Table 3.

**Table 3:** Evaluation criteria for the “step-up” approach for the treatment of infected necrotising pancreatitis

**Feasibility of step-up approach: 100%**	
**Resolution of the infection** ^**a**^ **: 80.5%**	
**Morbidity of the interventional procedures** *1. Complication during the procedure* - Endoscopy (stenting and necrosectomy): haemorrhage (6%)^b^ - Radiology: none - Surgical mini-invasive necrosectomy: none - Open surgery: none *2. Delayed complications (within one month following the procedure)* - Endoscopy: stent migration (29.5%); Gastric ulcerations without bleeding (9%) - Radiology: drain obstruction (16%) - Surgical mini-invasive necrosectomy: none - Open surgery: none^c^	
**Mortality at 6 months: 19.5%**	
**Long term sequelae** ^**d**^ - Exocrine pancreatic insufficiency: 27% - Endocrine pancreatic insufficiency: 45% - Pancreatic fistulae with pancreatic duct disconnection: 24% - Hernia: 3%	

a: According the composite criteria (resolution of OF and at least two of the following clinico-biological criteria: apyrexia, decrease of at least 50% of CRP serum level, decrease of at least 25% of total leucocytes count)

b: Requiring endoscopic haemostasis but no blood transfusion

c: The patients died at least one week after surgery but the cause of death was related to the evolution of infected necrosis and persistent sepsis (and not directly to the surgical procedure)

d: Evaluated on 33 patients

### Morbidity of intervention procedures

#### **Endoscopy**

The only immediate complication (LAMS placement) was mild haemorrhage. There were no complication during the necrosectomy sessions. When the stent was removed, only one complication occurred (3%), as a haemorrhage requiring the insertion of two haemostatic clips (no transfusion). We observed a migration of stents in 10 patients (29%) (1 plastic and 9 LAMS – diagnosis by CT-scan). Only one migration required surgery because of its impaction in the small bowel (LAMS). The others stent either migrated without complication (*n* = 5) or have been removed during dedicated endoscopic procedure (*n* = 3)(Table 3).

#### **Radiology**

There were no complications during radiological drainage procedures. Six drains (16%) drains had to be changed due to their obstruction. Only one patient had a haemorrhage (without haemodynamic instability or anaemia) with mild bleeding externalised by one of his drains (no pseudo-aneurysm at CT-scan) and the bleeding stopped spontaneously (Table 3).

#### **Surgery**

There were no immediate complications during either the six minimally invasive surgical necrosectomies nor during laparotomy necrosectomy surgeries.

### Six months mortality

Eight patients died representing a mortality rate of 19.5%. Four patients died after drainage: one patient by haemopericardium tamponade, two others by extensive mesenteric ischemia and one by septic shock with multiple OF. Four patients died after step 3 of invasive open surgical necrosectomy (7 to 15 days after surgery): 3 patients with persistent septic shock and multiple OF and one with extensive mesenteric ischemia and haemorrhage. Table 4 detailed the risk factors associated with death. The risk factors associated with death and statistically significant after multivariate analysis were: the presence of a SIRS at admission, the presence of one or more OFs before step 1, a fungal infection, the size of the largest collection ≥ 16 cm.

**Table 4:** Uni- and multivariate analysis of risk factors of death in infected necrotising pancreatitis patients treated in a step-up strategy

Variables *(Number of deaths at 6 months)*	Univariate analysis	Multivariate analysis
	OR (95% CI; *p)*	OR (95% CI; *p)*
pre-existing co-morbidity^a^		
yes: 19 *(4)* ; no: 22 *(4)*	1.16 (0.43–10.19; 0.40)	-
Referred from another centre^b^		
yes: 31 *(7)* ; no: 10 *(1)*	2.25 (0.31–16.2; 0.65)	
SIRS at admission		
yes: 19 *(7)* ; no: 22 *(1)*	**8.1 (1.09–60.1; 0.015)**	**12.25 (1.34-111.89; 0.026)**
One or multiple OF before the step-1		
yes: 17 *(6)* ; No: 24 *(2)*	**4.2 (1.01–18.5; 0.048)**	**6 (1.03–34.74; 0.046)**
Intensive care unit		
yes: 19 *(5)* ; no: 22 *(3)*	6.42 (0.52–7.03; 0.43)	
Biliary AP		
yes: 25 *(5)* ; no: 16 *(3)*	1.067 (0.29–3.86; 1.00)	
Fungal Infection		
yes: 15 *(6)* ; no: 26 *(2)*	**5.2 (1.19–22.5; 0.035)**	**7.99 (1.35–47.1; 0.022)**
Radiologic drainage		
yes: 18 *(2)* ; no: 23 *(6)*	0.42 (0.09–1.86; 0.42)	
Necrosectomy^c^ (Step-2)		
yes: 15 *(2)* ; no: 26 *(6)*	0.57 (0.13–2.51; 0.68)	
Necrosectomy by open laparotomy (step-3)		
yes: 4 *(4)* ; no: 37 *(4)*	**9.2 (3.6–23.3; 0.0007)**	^d^
Size of the collection ≥ 16 cm		
yes: 17 *(7)* ; no: 24 *(1)*	**9.8 (1.33–73.1; 0.0051)**	**16.1 (1.74-148.67; 0.014)**

OR: Odds ratio – CI: confidence interval – OF: organ failure

a: co-morbidities “cardio-vascular, pulmonary, renal and obesity”

b: patients referred from another hospital (primary or secondary centre)

c: minimally invasive endoscopic and/or surgical necrosectomy

d: no multivariate analysis due to the small number of events

### Long term follow-up

A total of 33 patients were followed-up after the step-up approach (until September 2022). The mean follow-up was 68.5 ± 24.2 months (median: 72 months – extremes: 10–107 months). Nine patients (27%) developed an irreversible exocrine pancreatic insufficiency. Fifteen patients developed or worsened a previous diabetes (45%). Eight patients had pancreatic fistula (24%): 6 internal fistulas with pancreatic duct disconnection and 2 external fistulas. For internal fistula, mostly associated with peri-pancreatic collections and/or pseudocysts, the management consisted in a prolonged exclusive artificial nutrition (enteral or parenteral) associated or not with endoscopic treatments (pancreatic stenting and/or collections drainage). External fistulas were treated with enteral nutrition associated with stable somatostatin analogues. One patient developed an abdominal hernia in place of a minimally invasive necrosectomy. We did not observe either recurrent AP or development of chronic pancreatitis.

## Discussion

Our study, conducted on a cohort of 41 successive patients, demonstrates that the step-up approach for the treatment of infected WON is highly feasible with little morbidity and no mortality from procedures performed alone or in combination. Only few studies have looked at the “step up” strategy as a whole. Beside the feasibility, this step-up approach displayed a clinical and biological efficacy in 80% of cases. We have also investigated the long-term sequels and morbidity which are completely acceptable regarding the severity of the disease.

To evaluate the efficacy of our protocol we applied a simple composite criterion that included new onset or persistence of OF and clinico-biological criteria of infection. On the whole, the simple endoscopic and/or drainage allowed to resolve near 50% of infected necrosis (as reported also by Rana et al. [22]). After this first step we also observed a significant decrease of key biological markers of infection (CRP level and white blood count) within the next 14 days. In addition, necrosectomy allowed to correctly treat 30% of patients.

Previous studies had included various criteria of evaluation such as “death, new or persistent organ failures, complications of the procedures … etc”. In the present study, we have added three simple clinico-biological criteria of infection to evaluate our step-up approach because: (i) all patients did not have necessarily an OF at time of first drainage; (ii) persistence of clinical-biological signs of infection may be the first signs of a possible occurrence of a subsequent OF. However, this composite criterion merit prospective (multicentric) evaluation to be validated.

In the present study the mortality at 6 months (19%) was similar to that observed in randomised studies evaluating minimally invasive treatments of infected WON, i.e. between 13 and 18% [7–10]. However, noteworthy that these results have never been described in the “real life”. The main causes of death were abdominal extension of necrosis with vascular damage and sepsis [7–12]. Mortality factors were classic, with the presence of a SIRS and several OF at onset of AP, a fungal infection and the size of the walled-off necrosis [5, 15]. Concerning the fungal infection, this is a frequent event that can occur (even in the absence of antibiotic therapy), with a major impact on prognosis [23]. A recent analysis included this infection as a factor in the failure of the minimally invasive step-up approach [24]. Preventive treatment is even recommended, but the cost/effectiveness ratio needs to be assessed [25]. On the whole, when applying this set-up strategy, the mortality rate for this type of patient should not exceed 15–20% (versus 35 to 40% formerly).

The long-term sequelae and morbidity have only been studied in three studies, including our own. The rate of exocrine insufficiency ranges from 29 to 61%, that of endocrine pancreatic insufficiency for 36 to 45% [26]. Fistulae, most often related to pancreatic disconnection (20 to 30%), are entirely treatable by resting the pancreas and endoscopic approach, which reduces the rate of fistulae and collections beyond 6 months. They do not cause secondary mortality, but may prolong hospital stays.

Concerning endoscopic procedures, our centre has decided to use LAMS (as soon as they were placed on the market), rather than plastic prostheses. LAMS have many advantages (compared to plastic prostheses), related to their large diameter (from 12 to 16 mm) and their design (flanges at both ends): possible evacuation of necrotic debris through the prosthesis with a lower risk of obstruction, placement of a naso-cystic drain or double-pig tail stents directly through the prosthesis and direct access to the necrotic cavity for necrosectomy sessions [27, 28].

Many studies have compared the different types of stents [29–32]. There is two randomised controlled trial comparing plastic prostheses and LAMS in the treatment of necrotic collections (infected or symptomatic) [31, 32]. All studies concluded that the two types of prostheses are equivalent in terms of technical success, effectiveness in resolving collections, adverse events, cost of treatment and length of hospitalisation. Only the duration of the procedures was shorter for metal prostheses.

Another technical aspect is the placement of concurrent double-pig tail stent through (coaxial) the LAMS that could avoid some complications and LAMS obstruction. Two randomised studies have different results and the advantage of this supplemental procedure remains to be more evaluated [33, 34]. In the same way we have placed in more than two third of our patients with LAMS a 10 F nasocystic drain at the same time followed by washing and drainage by gravity during at least 3 days. Whether these drains allowed to better drain the cavity and then avoid LAMS obstruction was not demonstrated but they were well tolerated [18].

The weaknesses of this work are that the analysis was retrospective, monocentric and a long inclusion period. Nevertheless, it is a real-life analysis with similar results to the few prospective studies [20, 24]. Our results therefore validate this step-up approach, and the latest large-scale analyses suggest that this minimally invasive approach is being applied more and more systematically [20]. Further surveys of practice need to be carried out, and the concept disseminated [35].

## Conclusions

The “step up” strategy is nowadays the strategy validated by several consensus conferences for the treatment of necrosis infection occurring in severe acute pancreatitis. While most studies have studied or compared the different techniques, the present work is original because it evaluates the “step up” approach as a whole and in the real life. Our observational study conducted in a tertiary centre allows us to conclude that the multidisciplinary “step up” approach is feasible with a clinico-biological efficacy on infection in 80% of cases and with an acceptable morbidity, mortality and long-term sequels. Nevertheless, some points of the strategy should be clarified to establish a clearer management algorithm. To date, there are no consensual clinical or biological or radiological criteria defining failure and leading to the next step in the step-up strategy as well as timing, sequence and number of necrosectomy sessions. In this sense, it would be interesting to establish a composite score or precise criteria for response or non-response.

## Data Availability

The data that support the findings of this study are available from the corresponding author upon reasonable request.

## Abbreviations

AP: Acute pancreatitis
OF: Organ failure
WON: Walled-off necrosis
SIRS: Systemic inflammatory response syndrome
EUS: Endoscopic ultrasound
LAM: Lumen-apposing metal stent
